# Profiles of volatiles in microalgae depend on the extraction and analytical methods

**DOI:** 10.3389/fbioe.2025.1589776

**Published:** 2025-04-25

**Authors:** Carla C. C. R. de Carvalho, Kilian Neves, Sebastião V. T. F. de Almeida, Pia Steinrücken, Dorinde M. M. Kleinegris

**Affiliations:** ^1^ Department of Bioengineering, iBB-Institute for Bioengineering and Biosciences, Instituto Superior Técnico, Universidade de Lisboa, Lisbon, Portugal; ^2^ Associate Laboratory I4HB—Institute for Health and Bioeconomy, Instituto Superior Técnico, Universidade de Lisboa, Lisbon, Portugal; ^3^ NORCE Climate and Environment - NORCE Norwegian Research Centre AS, Bergen, Norway; ^4^ Department of Biological Sciences, University of Bergen, Bergen, Norway

**Keywords:** VOCs, microalgae, cell disruption, volatile extraction, organic solvents, gas chromatography-mass spectrometry, GC-MS

## Abstract

**Introduction:**

The incorporation of microalgae in food products is dependent on their volatile profiles, which significantly influence their organoleptic characteristics and, consequently, consumer acceptance. However, microalgae contain a myriad of volatile compounds, and their precise impact on olfactory and gustatory perception is not easily inferred. Nonetheless, volatiles imparting a fish-like odour are generally considered undesirable. To develop enzyme processes or other methods targeting malodour compounds, they must be identified and quantified in the microalgal biomass. However, no standard method allowing the extraction of all volatiles is available.

**Methods:**

In the present study, the volatile profiles of the freshwater microalgal species *Chlorella vulgaris* and *Arthrospira platensis *and of the marine microalgal species *Microchloropsis gaditana*, *Tetraselmis chui*, and *Phaeodactylum tricornutum* were determined by gas chromatography.

**Results:**

A total of five fractions were obtained by sequential elution of increasingly polar solvents and different methods to break the cells were tested. Additionally, the lipid composition of each species was determined and compared.

**Discussion:**

The study clearly shows that extraction and analytical methods have a strong impact on the reported volatile and lipid profile of the cells.

## 1 Introduction

The application of microalgae in food products is highly dependent on their volatile profile, which influences their organoleptic properties and thus consumer acceptance. However, there are numerous volatile compounds present in microalgae and their impact on smell and taste is not easily inferred, although those contributing to a fish-like smell are usually undesired.

The number of published studies on the volatile composition of both microalgae and cyanobacteria is scarce, and some studies regarding the effect of strain selection, cultivation parameters and downstream techniques on their fishy smell and flavour do not provide data on the compounds involved. Additionally, some studies have shown that the extraction method will determine the profile of volatile and flavour compounds (Lafarge and Cayot, 2019; Salinas-Salazar et al., 2019; Nunes et al., 2023). Among the most used techniques are solvent extraction, including by environment-friendly solvents (König-Mattern et al., 2021) and deep eutectic solvents (Mehariya et al., 2021), high pressure homogenization (Magpusao et al., 2022), ultrasound-assisted extraction (Ferreira et al., 2016), solid-phase microextraction (Moran et al., 2022), and supercritical extraction (Michalak et al., 2015). Since the methods will affect the proportion of extracted compounds due to their physico-chemical properties, different studies will result in distinct volatile profiles and thus in the assessment of the sensory properties of a given microalga or cyanobacterium. Contributing to this is the low concentration and diverse types of compounds, the techniques chosen for sample preparation, analytical limitations due to sensitivity and resolution required, and lack of standardization methods, making their detection and quantification challenging (Jalili et al., 2020; Li, 2023).

In the European Union, a number of microalgae species were consumed as food or in food supplements prior to May 1997, namely, *Aphanizomenon flosaquae*, various strains of spirulina *(Arthrospira. platensis*, *Limnospira fusiformis*, *Arthrospira major*, *Arthrospira maxima* and *L. indica*), various strains of *Chlorella (Auxenochlorella protothecoides, Auxenochlorella pyrenoidosa, Chlorella sorokiniana, Chlorella vulgaris, Jaagichlorella luteoviridis*, *Heterochlorella luteoviridis*, *Parachlorella kessleri*, and *Chlorella emersonii*), *Scenedesmus vacuolatus*, and *Dunaliella salina*. Since the implementation of the Novel Food Regulation (EU) No 2015/2283, DHA from *Ulkenia* sp., astaxanthin from *Haematococcus pluvialis*, oil from *Schizochytrium* sp., the diatom *Odontella aurita*, and the microalgae *Ulkenia* sp., *Euglena gracilis*, and *Tetraselmis chui* have been added to the list (EU, 2015; Efsa Panel on Nutrition et al., 2020). *Microchloropsis gaditana* is a good producer of omega-3 fatty acids, presenting a good potential as an eicosapentaenoic acid source for both human consumption and for animal feed (Camacho-Rodríguez et al., 2014; Molino et al., 2019). Similarly, *P. tricornutum* biomass is a promising candidate for the production of lipids and nutraceuticals due to the high content in lipids and fucoxanthin (Celi et al., 2022; Elshobary et al., 2025). Although there is a recognized nutritional value of microalgal biomass, namely, in proteins, polyunsaturated fatty acids, antioxidants, vitamins, minerals and carotenoids, the general consumer may avoid their consumption due to negative perception related to the colour and smell (Nunes et al., 2023; Olsen et al., 2024).

To improve the organoleptic properties of microalgae, it is important to determine their volatile profile and impact of different treatments such as drying and bead milling. Standard methods allowing the comparison of volatile profiles after, treatment of the biomass, to understand their impact on the sensory properties and on consumer acceptance are still lacking. The aim of the present work was thus to determine the composition of volatile compounds of freshwater and marine microalgal species. To determine the full extent of compounds, organic solvents of different polarities and miscibilities were used following cell wall disruption by freeze-thaw cycles, disruption with glass beads, or osmotic shock. Simultaneous extraction during osmotic shock was also tested. Solid phase extraction (SPE) techniques allowed the separation of volatile compounds into fractions which improved their identification by gas chromatography-mass spectrometry. The fatty acid profiles of the cells were also determined. The different methods should allow the determination of nearly all compounds present in the microalgal biomass.

## 2 Materials and methods

### 2.1 Biomass production

Freshwater microalgal species *C. vulgaris* (NIVA-CHL108) and *Arthrospira platensis* (NIVA-CYA 428) were obtained from the Norwegian Culture Collection of Algae (NORCCA). The marine microalgal species *Microchloropsis gaditana* (CCMP526) was acquired from Bigelow NMCA, and *T. chui* (UTEX LB232) and *Phaeodactylum tricornutum* (UTEX 640) from the UTEX culture collection.

Each microalgal species was maintained as stock culture in 15 mL glass tubes at 15°C and an irradiance of 50 μmol m^−2^ s^−1^ (Light:Dark cycle of 16:8 h). Freshwater strains (*C. vulgaris* and *A. platensis*) were grown in modified BBM medium, containing (in mM): NaNO_3_, 12.47; KH_2_PO_4_, 1.29; K_2_HPO_4_, 0.43; CaCl_2_·2H_2_O 0.17; MgSO_4_·7H_2_O, 0.30; NaCl, 0.43; B, 0.031; Cu, 0.002; Fe, 0.043, Mn, 0.017; Mo, 0.001, Zn, 0.008; Vitamin B1, 0.29·10^−3^; Vitamin B12, 0.37·10^−5^; Biotin 0.21·10^−4^. Marine strains (*P. tricornutum*, *M. gaditana,* and *T. chui*), were cultivated in NORCE medium (Steinrücken et al., 2023), prepared with sterilized natural seawater (35 ppt) enriched with nutrients in the following concentrations (mM): NaNO_3_, 12.47; KH_2_PO_4_, 0.88; B, 0.031; Cu, 0.002; Fe, 0.043, Mn, 0.017; Mo, 0.001, Zn, 0.008. For biomass production, cultures were upscaled to four 300 mL bubble column photobioreactors (24°C, 100 μmol m^-2^ s^-1^, aerated with 0.2 μm filtered and 1% CO_2_ enriched air), and subsequently to a 25 L tubular photobioreactor (PBR). For *T. chui*, *P. tricornutum*, *C. vulgaris* and *A. platensis*, biomass was directly harvested from the 25 L PBR while for *M. gaditana*, the culture was subsequently transferred to the National Algaepilot Mongstad.

The 25 L PBR (Lgem Lab-25) consisted of a vertical tubular glass helix (32 × 2.2 mm) with 12 windings through which the culture was circulated by airflow (0.2 µm filtrated). The reactor was placed in a temperature-controlled room to maintain a constant temperature of 23.0°C ± 0.5°C (26°C for *A. platensis*). The pH was maintained between 7.8 and 8 (8.8 and for 9.0 *A. platensis*) through controlled pulse-wise injection of 100% CO_2_, and irradiance was provided by LED light panels, placed on both sides of the reactor. The light intensity was increased gradually with the increase in biomass concentration from 30 to 550 μmol m^−2^ s^−1^. Cultures were grown in BBM or NORCE medium as described above until biomass concentrations of 4–7 g L^-1^ were reached. Nitrate concentration was monitored regularly with nitrate test strips (Quantofix), and additional nutrients were added when the nitrate concentration in the culture decreased. For *T. chui*, *P. tricornutum*, *C. vulgaris* and *A. platensis*, biomass was collected and dewatered by filtration (Vibro-lab3500, SANI membranes) and the obtained slurry was subsampled 6x into falcon tubes for trait analysis (approximately 1 g dried biomass per tube) and frozen directly. Three falcon tubes were kept frozen (frozen paste) while 3 tubes were then freeze-dried (freeze-dried biomass). All samples were stored at −20°C until shipment to Portugal and were shipped frozen.

Samples for *M. gaditana* biomass were taken from the ongoing production at the National Algaepilot Mongstad, in Norway (60.803230, 5.026794), where *M. gaditana* biomass was produced in various batches during 12 April – 17 July 2022 in one 250L and four 750L tubular photobioreactors (Lgem, Netherlands), located in a greenhouse. The production process involved a fed-batch approach, with reactors being harvested twice a week (50%–90% of the culture volume) and replenished with fresh seawater and nutrients. Illumination was provided by natural light, and additional artificial illumination (EAX 170W LED lights, Evolys AS, Norway) with an average incident irradiance of 220 μmol m^−2^ s^−1^. The pH was maintained at 7.8 by on-demand CO_2_ addition, and temperatures were kept between 15°C and 35°C by cooling through heat exchangers and heating through heaters inside the greenhouse. Mixing was provided by both liquid pump and air pump, resulting in a culture velocity of approximately 0.3 m s^−1^. The biomass was harvested and dewatered by spiral plate centrifugation (Evodos 25, Evodos BV, Netherlands), resulting in a paste of approximately 25% dry weight. This paste was vacuum packed in bags of approximately 1.5 kg paste and stored at −20°C until further processing (bead milling and spray drying). From one harvest (13 July 2022), fresh paste was subsampled in six falcon tubes for trait analysis (approximately 1 g dried biomass per tube) and frozen directly. Three falcon tubes were kept frozen (frozen paste) while 3 tubes were then freeze-dried (freeze-dried biomass). All samples were stored at −20°C until shipment to Portugal and were shipped frozen.

For bead milling, the in this period total produced (approximately 265 kg) vacuum-packed and frozen biomass paste (25% dry matter) was thawed overnight at 4°C, pooled and subsequently diluted to approximately 18% dry matter using tap water. Additionally, 500 ppm tocopherols (dry matter basis) was added to the biomass (Grindox 1032, water-dispersible 20% tocopherol blend with emulsifiers). Bead milling was performed using a Wab Dyno-mill Multi-lab bead, 0.3 mm Zirconia beads, a 1.4-L chamber, and standard agitator discs. The milling process was operated at 2865 rpm with a flow rate of 5.6 kg/h and tap water was used for cooling. The disrupted biomass had a temperature of approximately 26°C when exiting the chamber and was collected in 1.6 kg portions and instantly vacuum-packed and frozen. For spray-drying, the frozen, bead-milled biomass was thawed again overnight at 4°C and spray-dried on a GEA-Niro Standard P-6,3 spray-dryer, equipped with a rotary atomizer, at an inlet temperature of 212°C–219°C and an outlet temperature of 90°C–93°C. From this spray-dried biomass, biomass was subsampled in 3 falcon tubes for trait analysis (approximately 1 g dried biomass per tube). All samples were stored at −20°C until being shipped to Portugal and were shipped frozen.

### 2.2 Extraction of volatiles

Total volatiles were extracted with solvents with different polarities, namely: the water miscible *iso*-propanol (Fisher Scientific, Loughborough, United Kingdom), ethanol absolute (Panreac, Barcelona, Spain), acetonitrile (Merck, Darmstadt, Germany), and acetone (Sigma-Aldrich, St. Louis, United States of America); and, the water immiscible ethyl acetate, *n*-hexane, and methyl *tert*-butyl ether (all from Sigma-Aldrich).

To improve extraction of volatile compounds by the solvents, cell wall disruption was assessed using the following methods: freeze-thaw cycles; disruption with glass beads; and osmotic shock. Freeze-thaw cycles were done by freezing the cells at −16°C followed by thaw at room temperature for 5 consecutive cycles. Disruption of the cells was also promoted using 0.1 mm glass beads and stirring 3 times at 2850 rpm during 3 min in a Diruptor Genie, both from Scientific Industries, Inc. The osmotic shock was promoted by placing the algal biomass in a 15% NaCl solution. After 15 min, the suspension was centrifuged at 10,000 × *g* for 3 min, the supernatant was discharged, and milli-Q water was added to the pellet to disrupt the cells. After centrifugation under the same conditions, the supernatant was discharged. Additionally, VOC extraction was tested by simultaneous extraction and osmotic shock for water immiscible solvents, and by extraction after osmotic shock for water miscible solvents. The extracted phase containing the VOCs was collected after centrifugation and analysed by gas chromatography-mass spectrometry (GC-MS). Samples were extracted in triplicate.

### 2.3 Fractioning of compounds

To improve the separation and identification of volatile compounds present in the microalgae biomass, they were separated into fractions obtained by solid phase extraction (SPE). The fraction of compounds present in the microalgal extract was adapted from previously published studies by Tava et al. (1991) and Rzama et al. (1995). In the present study, between 50 and 70 mg of algal biomass were extracted with 1 mL of methyl *tert*-butyl ether (MTBE). Following vigourous shaking, the mixture was centrifuged for 3 min at 10,000 × *g* and the MTBE layer was placed in glass vials closed with Teflon caps until it was loaded into the SPE columns. The different compounds were separated using 500 mg/6 mL Strata^®^ SI-1 silica (55 μm, 70A) SPE columns (Phenomenex^®^, Torrance, CA, United States of America). A total of 5 fractions were obtained by sequential elution of increasingly polar mixtures. The first fraction (F1) was the collection of the 1 mL of MTBE that eluted following the application of the sample, corresponding to non-adsorbed compounds which eluted with the MTBE. The following fractions were the result of the elution of 1 mL of each of the following solvents and mixtures: *n*-hexane (F2); MTBE (F3); MTBE:methanol (9:1, v/v) (F4); MTBE:methanol:acetic acid (45:4:1, v/v) (F5). Samples were extracted in triplicate.

### 2.4 Lipid profile of microalgae

The analysis of the fatty acid (FA) profile of the cells was done after extraction and methylation of the FA to fatty acid methyl esters (FAMEs), using the Instant FAME™ method from MIDI (MIDI, Inc., Newark, DE, United States) (Kunitsky et al., 2006). FAMEs were analysed on an Agilent Technologies 6890N gas chromatograph with a flame detector (GC-FID), and a 7683 B series injector, with hydrogen as carrier gas. The compounds were separated by a 25 m Agilent J&W Ultra two capillary column. The FAME profile was determined using the Sherlock^®^ software package (version 6.2) using the PLFAD1 method. The FAMEs were identified by the MIDI software and also by injection of samples on an Agilent 7820A gas chromatograph with an Agilent 5977E quadrupole MS detector equipped with a 25 m Agilent J&W Ultra two capillary column. The temperature conditions were the same as the PLFAD1 method from MIDI.

### 2.5 Analysis of volatiles

The volatile compounds were analysed on an Agilent 7820A gas chromatograph with an Agilent 5977E quadrupole MS detector equipped with a 25 m Agilent J&W Ultra two capillary column, with helium as carrier gas. The quantification of trimethylamine was carried out with the following temperature program in the GC oven: initial temperature at 40°C for 2.5 min, followed by an increase of 15°C/min until 240°C. The injection was made in splitless mode with the injector at 200°C, whilst the MSD transfer line was at 280°C. All samples containing volatiles were analysed by two oven temperature programs: one starting at 40°C and finishing at 150°C at a 7°C/min rate; the other starting at 40°C and finishing at 240°C at a rate of 15°C/min. The injector was at 200°C but in the former program the injection was made in splitless mode, whilst in the latter the injection was done in split mode at a 20:1 split ratio. The MSD transfer line was at 280°C in both programs. Peak identification was performed using the Qualitative Analysis B.07.00 software, part of the MassHunter Workstation from Agilent, by comparison to the NIST mass spectral library version 2.2 and also by injection of standard compounds. Peak quantification was done by the Quantitative Analysis B.07.00 software, part of the same MassHunter Workstation.

## 3 Results and discussion

### 3.1 Microalgal total volatile composition

**FIGURE 1:**
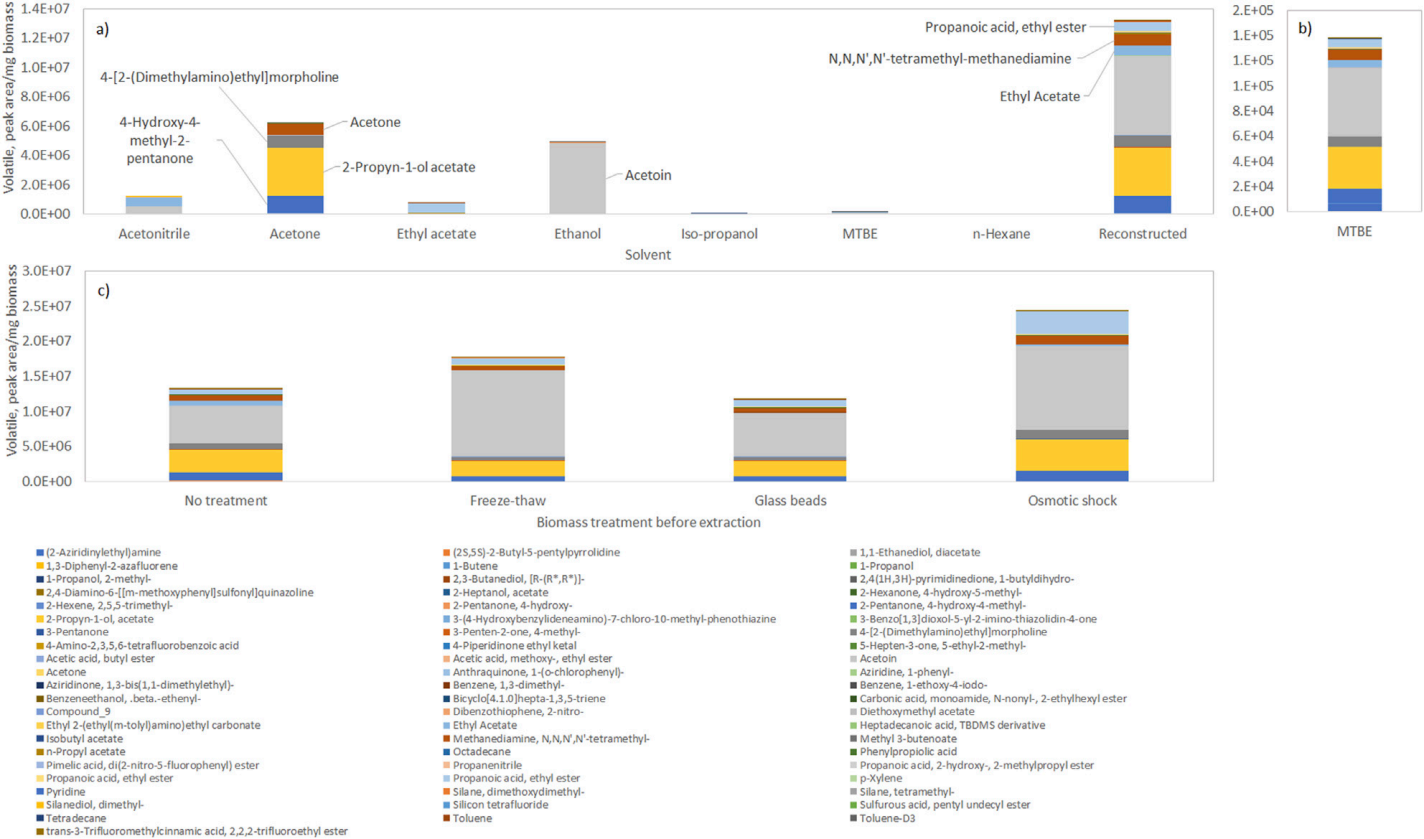
Profile of volatile compounds extracted from frozen biomass of *Tetraselmis chui* with solvents with different polarity (with solvent polarity decreasing from left to right), and reconstructed total volatile profile. **(a)** Composition in volatiles in each solvent. **(b)** Composition in volatiles in MTBE. **(c)** Reconstructed total volatile profile after extraction of volatiles by the solvents after submitting the biomass to freeze-thaw cycles, vigorous shaking with glass beads, or osmotic shock. Data shown are average values of three independent samples.

To determine the composition of volatile organic compounds in microalgae, several solvents were tested to extract the compounds, namely, by decreasing polarity: the water miscible acetonitrile (Polarity index, P’ = 5.8), acetone (P’ = 5.1) (absolute) ethanol (P’ = 4.3), and *iso*-propanol (P’ = 3.9); and the water immiscible ethyl acetate (P’ = 4.4), MTBE (P’ = 2.5), and *n-*hexane (P’ = 0.1) (Figure 1a). The solvents were chosen based on their physicochemical properties, in particular polarity, in order to extract the greatest diversity of compounds that could be encompassed in the biomass for further characterization. Polar solvents are more effective at extracting polar compounds, due to the formation of hydrogen bonds and dipole-dipole interactions, whilst non-polar solvents extract non-polar hydrophobic compounds through van der Waals forces. Acetone, ethanol, acetonitrile and ethyl acetate, the most polar solvents, allowed the removal of the highest amount of compounds from dried biomass of *T. chui* (Figure 1a).

By normalizing the amount of compounds present, using the amount of biomass extracted, it was possible to reconstruct the total extractable volatile profile of the cells by using the data collected with all solvents (Figure 1a), “Reconstructed”). The compounds present in the largest amount in dried *T. chui* cells were the following: acetoin (47.6%); 2-propyn-1-ol acetate (22.6%); 4-hydroxy-4-methyl-2-pentanone (7.7%); ethyl propionate (7.4%); *N*,*N*,*N′*,*N′*-tetramethyl-methanediamine (5.9%); and 4-[2-(dimethylamino)ethyl]morpholine (5.9%). Although the amount of volatiles extracted by MTBE was low, in comparison with other solvents, this solvent allowed a profile of compounds similar to the one obtained after “reconstruction” (Figure 1b). The amount of volatiles extracted could be enhanced by subjecting the algal biomass to freeze-thaw cycles or osmotic shock prior to the solvent extraction (Figure 1c). The former allowed a 25% increase in the amount of volatiles extracted whilst the osmotic shock was more efficient resulting in a 77% increase in the amount of extracted volatiles by the solvents. A 2.2-fold increase in the amount of acetoin was observed after both freeze-thaw cycles and osmotic shock when compared to cells simply extracted by organic solvents. Cell disruption by vigorous shaking with glass beads, on the other hand, did not result in increased extraction yields. The freeze-thaw method is simple and cost-effective, but multiple cycles may be required for effective disruption, which can be time-consuming and may be inefficient for species with rigid cell walls. Cell disruption with glass beads is usually highly effective for breaking rigid cell walls and may be scaled up for industrial applications, but specialized equipment is necessary and heat is generated during operation, which may degrade heat-sensitive compounds. In the present study, the amount of volatiles extracted from *T. chui* cells using glass beads was similar to the amount obtained when no specific disruption method was used (Figure 1c). The osmotic shock is a method that allows the disruption of the cells without mechanical damage, which is usually effective for species with less rigid cell walls, but finding the precise osmotic conditions to break the cells may be challenging. In the present study, the osmotic shock method allowed the extraction of the largest amount of volatiles/mg of extracted biomass (Figure 1c), which could be due to the fact that *T. chui* is a marine alga.

**FIGURE 2:**
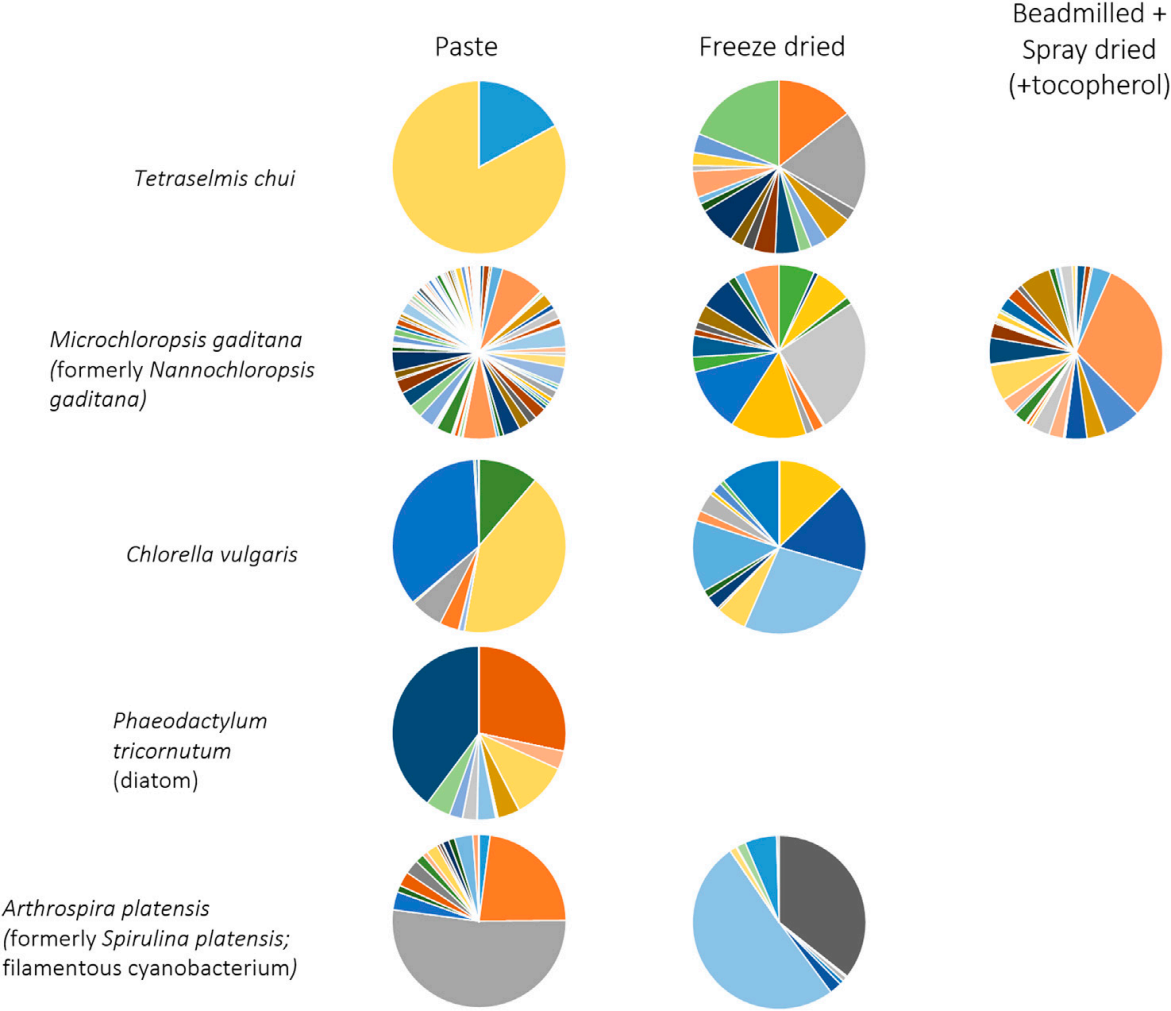
Volatile profile of microalgae extracted with MTBE from biomass paste, freeze dried biomass and biomass that was beadmilled and spray dried in the presence of tocopherol. Compounds and colours listed in Figure 1. Data shown are average values of three independent samples.

The effect of treating the algal biomass by (i) freeze drying and (ii) beadmilling and spray drying, on the volatile profile of the microalgae, was also assessed and compared to the extraction made directly from concentrated biomass paste. Distinct volatile profiles could be observed after the different treatments, even for the same solvent (Figure 2; biomass extracted with MTBE). Other studies have shown that post-harvest processing techniques can alter the volatile profile of algae, and thus their aroma and flavour (Coleman et al., 2023; Urlass et al., 2023). Spray drying was found to reduce the intensity and the grassy odour of *Nannocloropsis* sp. cells whilst high pressure homogenization resulted in intense grassy and fishy oil odours (Coleman et al., 2023). While the former method was linked to a decrease in esters, saturated alcohols and dimethyl sulfide, the latter was related to high amounts of fatty acid-derived unsaturated aldehydes, ketones and alcohols. In the present study, MTBE extracted a much larger number of compounds from freeze dried *T. chui* cells than from biomass paste, but the contrary was observed for *M. gaditana* (Figure 2). Several volatile compounds were observed when the *M. gaditana* cells were beadmilled and spray dried in the presence of tocopherol. Additionally, different profiles were observed following MTBE extraction of freeze-dried cells of *C. vulgaris* and of the cyanobacterium *A. platensis* when compared to the volatile profile of biomass paste (Figure 2).

### 3.2 Fractioning of volatiles into classes

Fractionation of volatiles was based on previously published studies (Tava et al., 1991; Rzama et al., 1995) but the solvents were changed. In the published works, the hydrocarbons were eluted with pentane, the polar fractions were eluted with diethyl ether followed by a solution of diethyl ether-methanol (9:1, v/v), whilst the acidic fraction was eluted with a 45:4:1 (v/v) mixture of diethyl ether-methanol-acetic acid. In the present study, diethyl ether was replaced by MTBE which is a relatively inexpensive solvent with similar properties, but less volatile, less flammable, and much less prone to form peroxides. The remaining solvents were added in the suggested proportions.

**FIGURE 3:**
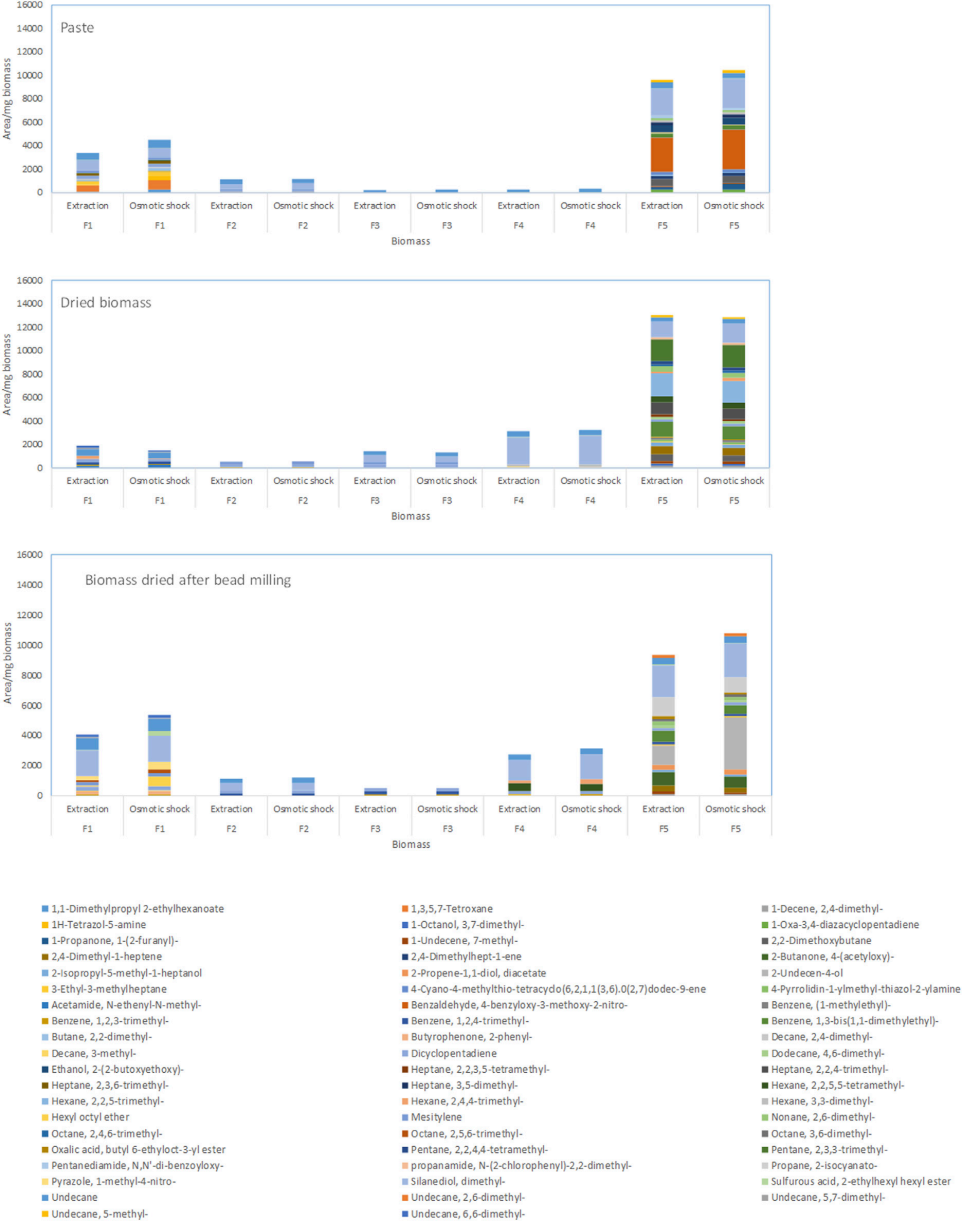
Composition of the fractions obtained by SPE of the biomass of *Microchloropsis gaditana* in paste, dried, and dried after bead milling. Data shown are average values of three independent samples.

In general, larger amounts of volatile compounds could be extracted after breaking the cells by osmotic shock in comparison to biomass directly extracted with only MTBE (Figure 3 for *M. gaditana*). Fractions 1 and 5 presented the largest amounts of compounds, with the main components of the biomass of *M. gaditana* being hydrocarbons and alkanols (Figure 3). Additionally, it was observed that the composition of the 5 fractions obtained from the biomass in paste, after drying, and after bead milling and drying, was different. Similar results were obtained for the remaining species.

**FIGURE 4:**
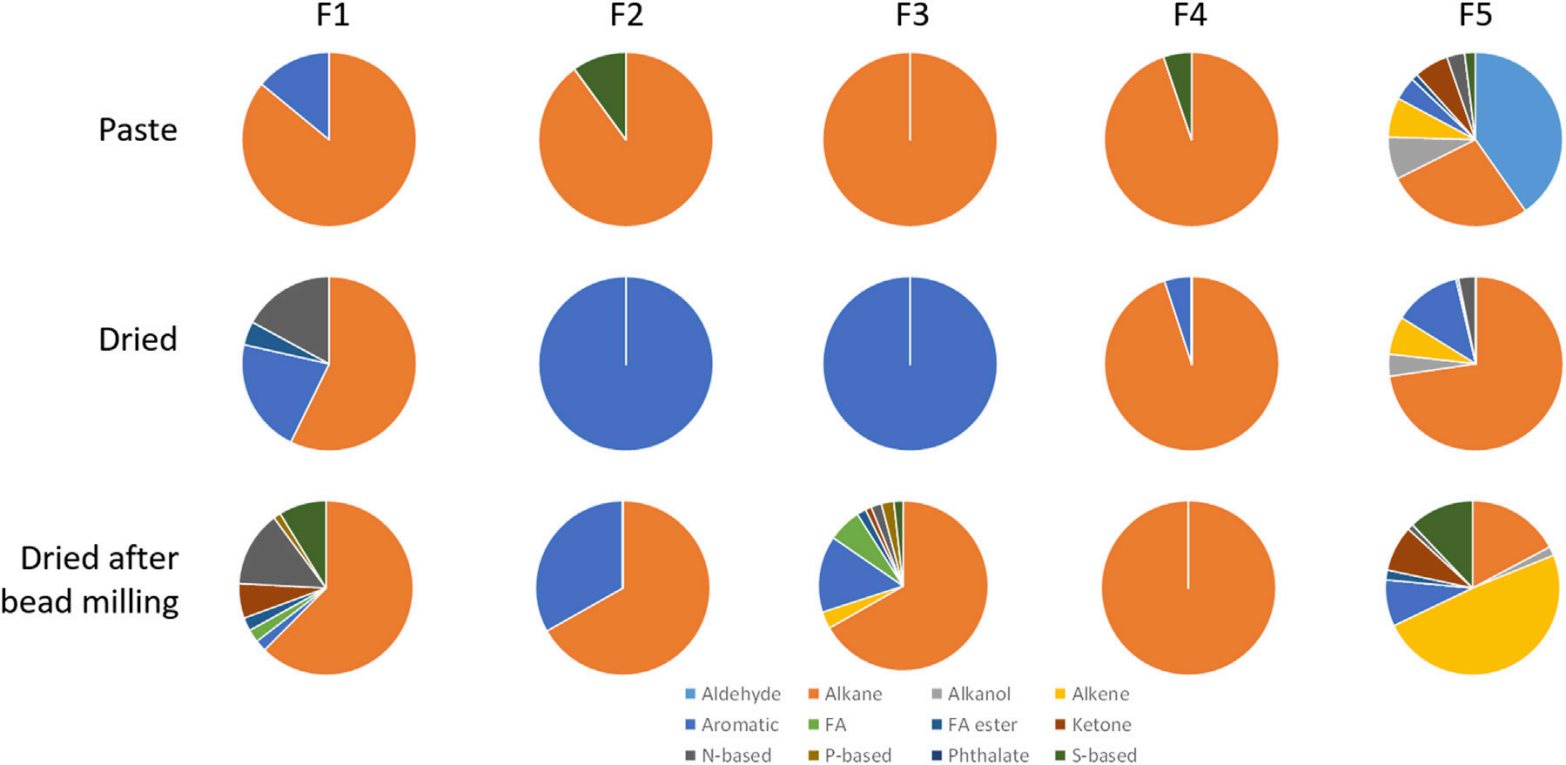
Volatile profiles of the 5 fractions obtained by SPE from *Microchloropsis gaditana* biomass paste, freeze dried biomass, and biomass that was beadmilled and spray dried in the presence of tocopherol, classified by their chemical structure. Data shown are average values of three independent samples.

When the compounds extracted from the *M. gaditana* biomass in paste and from dried biomass after bead milling were fractioned, the first four fractions contained mainly alkanes, corresponding to 62%–100% of total volatiles (Figure 4). Fraction 1 and 4 of dried *M. gaditana* biomass contained, respectively, 57% and 95% of alkanes. However, fractions 2 and 3 of the dried biomass only contained aromatic compounds. The *M. gaditana* biomass that was dried after bead milling presented ca. 65% alkanes, in the first 3 fractions, but significant amounts of aromatic, N-based and S-based volatiles could also be found. Nevertheless, fraction 5 contained different volatile classes in significant amounts, regardless of the state of the biomass, including alkanes, alkenes, aldehydes, and compounds containing sulfur and nitrogen.

**FIGURE 5:**
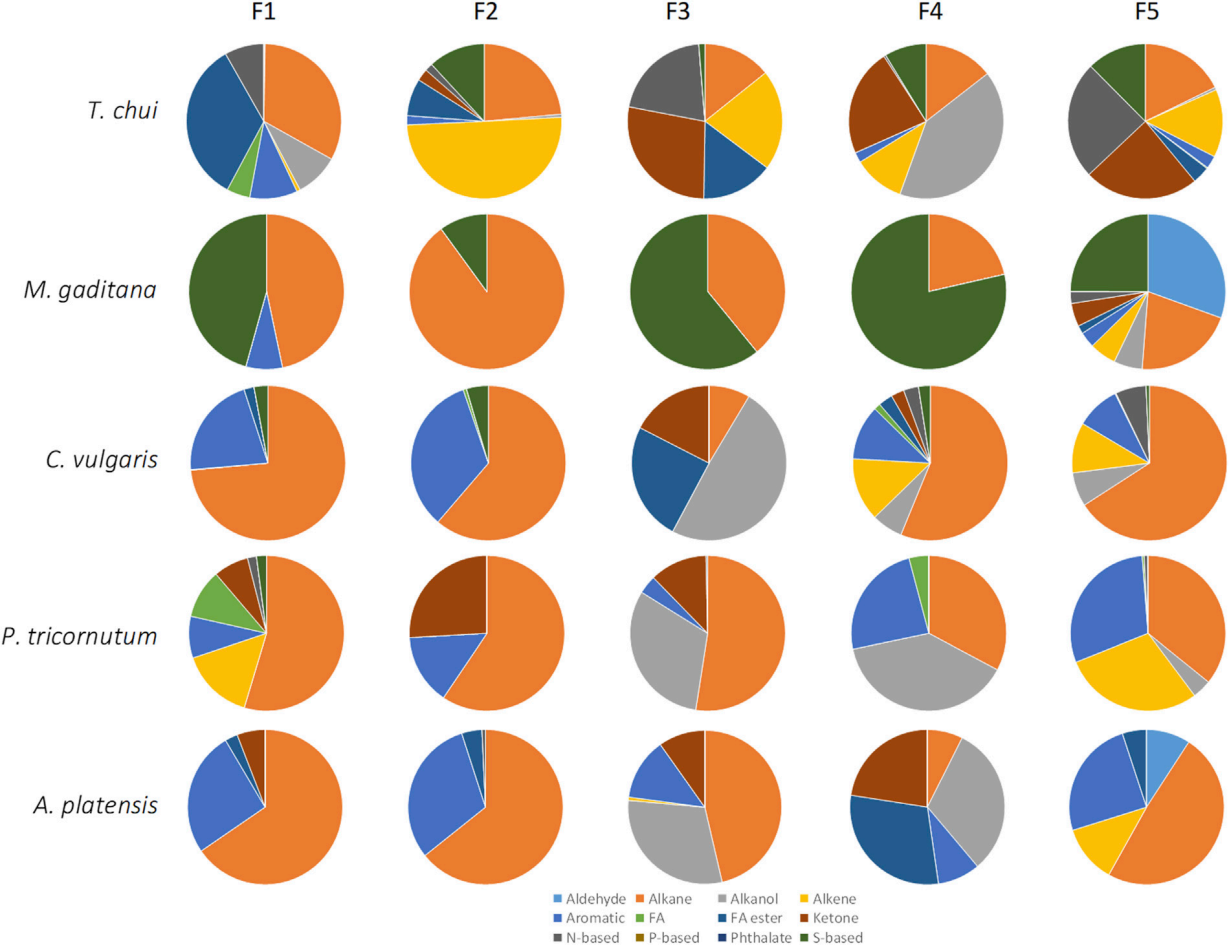
Volatile profiles of the 5 fractions obtained by SPE from the biomass paste of *Tetraselmis chui*, *Microchloropsis gaditana*, *Chlorella vulgaris*, *P. tricornutum*, and *A. platensis*, classified by their chemical structure. Data shown are average values of three independent samples.

In general, all tested microalgae biomass in paste contained significant amounts of alkanes in all fractions, with a minimum of 7.4% being observed in fraction F4 for *A. platensis* and a maximum of 89.9% being observed on fraction F2 for *M. gaditana* (Figure 5). Contrarily to what was observed with *M. gaditana*, where most of the volatile compounds were detected in F5, in the other microalgae the number of different compounds observed was balanced between fractions. In *T. chui*, F1 contained 33% alkanes and 34% esters of fatty acids, whilst F2 contained 50% alkenes and F4 contained 41% alkanols. But ca. 25% of F3, F4 and F5 were ketones, and around 20% were N-based compounds in F3 and F5. Among the compounds present in larger amounts in the different fractions were 2,6,11-trimethyl-dodecane (which has a gasoline-like odour), 2-cyclohexen-1-ol (rubber odour), acetoin (buttery odour), and dimethoxymethane also known as methylal (odour similar to chloroform). This supports the complexity of the final smell of each microalga.

*Chlorella vulgaris*, *P. tricurnutum* and *A. platensis* contained fractions with over 30% of alkanols, ca. 30% aromatic compounds, over 20% ketones, and low content of N- and S-based compounds, contrarily to *T. chui* and *M. gaditana* (Figure 5). In *C. vulgaris* compounds such as 1-butyl 2-isobutyl phthalate, neophytadiene (tobacco odour), methyl isobutyl ketone (spicy-fruity smell), and (*S*)-methyl oxirane (ether-like odour), were present (data not shown). In the paste of *P. tricornutum*, among the compounds present in larger amounts in the fractions were acid hexadecenoic (mild, fatty odour), dibutyl phthalate (slight aromatic odour), methyl isobutyl ketone (sweet, fruity odour), *N*-(1-ethylpentylidene)-methylamine, and 2-methyl-hex-2-yn-4-one. In *A. plastensis,* the volatile compounds present in larger amounts included undecane (+)-*N*-benzyl-alpha-methyl-*N*-nitrosobenzylamine, 2-hexanol (winey, terpenic odour), methyl-3-butenoate (fruity odour), and 1,3-bis(1,2-dimethylethyl)-benzene. The presence of compounds such as those listed demonstrates the complexity of the volatiles produced by these species.

### 3.3 Lipid composition

Lipids are one of the major constituents of microalgae biomass, reaching 5%–60% of the cell dry weight, being involved in cell structure and energy storage (de Carvalho and Caramujo, 2018; Morales et al., 2021). Additionally, several volatile compounds are produced from fatty acids: short-chain aldehydes and linear ketones are formed from fatty acid oxidation (Rzama et al., 1995; Boonprab et al., 2003); branched hydrocarbons from the oxidation of branched-chain fatty acids (Manning, 2022); alcohols are mainly formed as result of the secondary decomposition of hydroperoxides of fatty acids (Moran et al., 2022).

**FIGURE 6:**
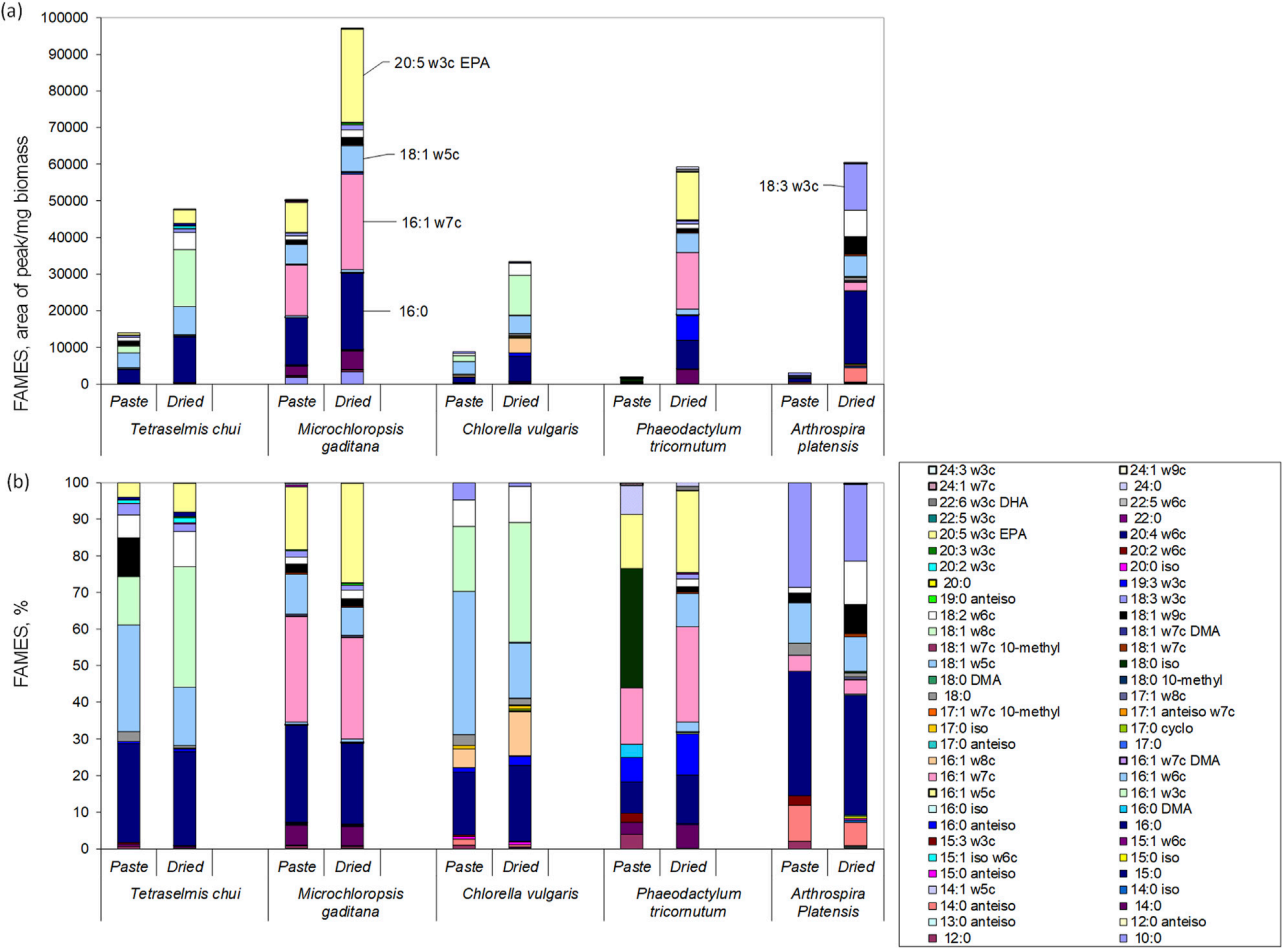
Lipid profiles of the wet paste and dried biomass of *T. chui, M. gaditana, C. vulgaris*, *P. tricornutum*, and *A. platensis*: **(a)** area of fatty acid per mg of biomass; **(b)** percentage of each fatty acid in the lipid fraction. Data shown are average values of three independent samples.

In the present study, the amount of lipids that could be extracted from the microalgal biomass was significantly larger when the dried biomass was used in comparison to wet paste (Figure 6a). Nevertheless, the fatty acid composition of both wet and dried biomass was similar for each of the 5 species analysed (Figure 6b). Both *T. chui* and *C. vulgaris* presented large amounts of 18:1w5c, 18:1w8c and 16:0 (Figure 6). In *M. gaditana,* the fatty acids with largest content were 16:1w7, 16:0 and 20:5w3c, whilst in *P. tricornutum* the fatty acids in largest amount were 18:0 iso, 16:1w6c and 20:5w3c. In *A. platensis*, the largest contents were observed for the fatty acids 16:0, 18:3w3c and 18:1w5c.

Regarding the content in polyunsaturated fatty acids, in *A. platensis* reached ca. 33% of total fatty acids, whilst in *M. gaditana* and *P. tricornutum* it was 31% and 27%, respectively (Figure 6). Lower contents of polyunsaturated fatty acids were observed in *T. chui* and *C. vulgaris* with 23% and 12%, respectively. Three of the most interesting long chain polyunsaturated fatty acids known for providing health benefits in humans are *α*-linolenic acid (ALA, 18:3ω3), docosahexaenoic acid (DHA, 22:6ω3), and eicosapentaenoic acid (EPA, 20:5ω3) (de Carvalho and Caramujo, 2018; Manson et al., 2019; Jesionowska et al., 2023). Under the culture conditions tested, *T. chui*, *M. gaditana*, and *P. tricornutum* produced the largest amounts of EPA (Figure 6). Additionally, the amount of EPA extracted was between 1.5 and 1.9-fold higher when the biomass of these species was dried in comparison to wet biomass. The highest content in EPA was achieved with dried *M. gaditana*, reaching 27.1% of total lipids. On the other hand, DHA was only produced by *P. tricornutum*, reaching 1% of lipids extracted from dried biomass. The amount of ALA varied among species and, curiously, was higher when wet biomass was extracted in comparison to dried biomass. In *T. chui*, ALA was 2.98% and 1.9% of total extracted lipids in wet paste and dried biomass, respectively, whilst in *C. vulgaris* was 4.8 and 1.0. The largest amount was produced by *A. platensis*, reaching 28.6% and 21.0% in wet paste and dried biomass, respectively. Since the lipid content is dependent on the environmental conditions, cultivation media, and as shown, extraction method, a direct comparison with published data is thus difficult. Nevertheless, other studies have shown that *A. platensis* cells only produce EPA and DHA in very low amounts (Diraman et al., 2009), whilst *M. gaditana* has been known to be a EPA producer (Ma et al., 2016).

As mentioned at the beginning of this section, lipids may be precursors to volatile compounds through processes such as autoxidation, photooxidation, and enzymatic oxidation. For example, PUFAs are highly prone to oxidation. Oxidation of linoleic acid results in the production of 9- and 13-hydroperoxides, and in the generation of various volatiles including 2,4-decadienal, 2-octenal, and hexanal, due to the cleavage of the C–O bond by alkoxy radicals derived from these hydroperoxides (Shahidi and Hossain, 2022). Thus during microalgae processing and storage, lipids may contribute to distinct aromas from fresh to rancid smells.

### 3.4 Extraction of trimethylamine

**FIGURE 7:**
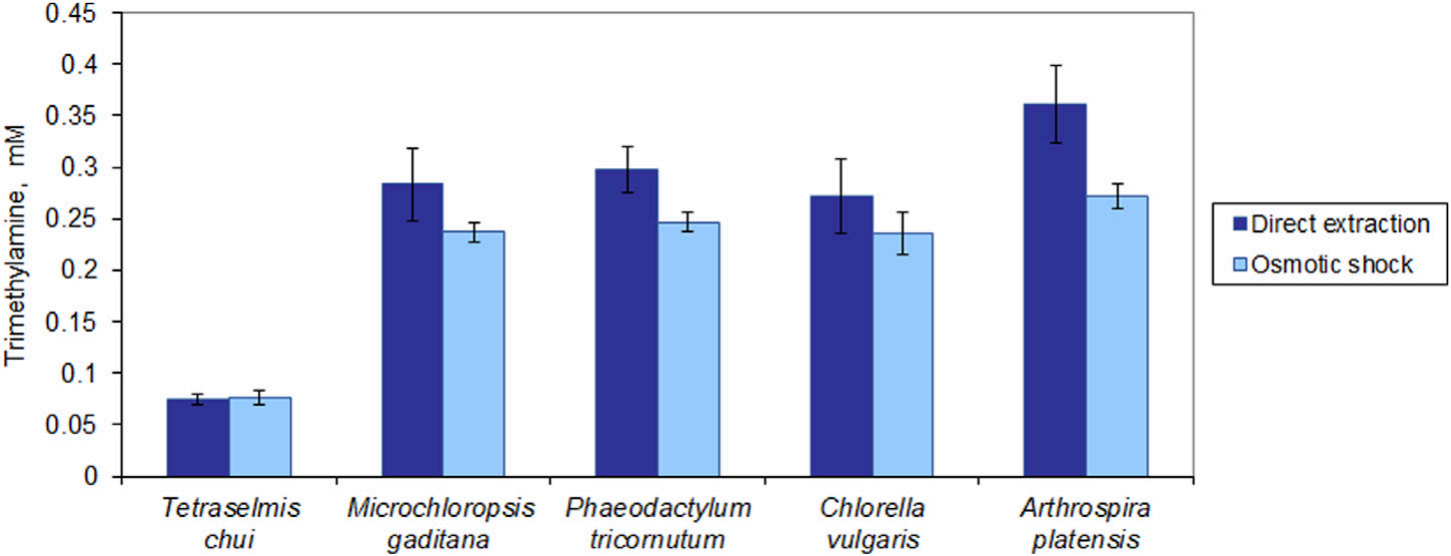
Trimethylamine (TMA) extracted from dried microalgal biomass. Extraction of TMA was carried out by direct extraction with MTBE and following rupture of the cells by osmotic shock. The data was normalized by the amount of biomass extracted. The data represents the average of 3 independent assays ±standard deviation.

The fishy odour in microalgal biomass may be the result of aldehydes (e.g., hexanal, heptanal, and nonanal), alcohols such as 1-octen-3-ol, sulfur-containing compounds such as dimethyl sulfide, and amines (Liu et al., 2024). Among the latter is trimethylamine (TMA). This compound has a strong fishy odour with a reported threshold between 0.00021 and 0.00058 ppm, which corresponds to 3.6 × 10^−9^ and 9.8 × 10^−9^ M, respectively (Boraphech and Thiravetyan, 2015). All tested strains presented a much larger amount of TMA than this threshold (Figure 7). In *T. chui*, TMA concentration was ca. 0.07 mM but in *M. gaditana*, *P. tricornutum* and *C. vulgaris* it attained ca. 0.28 mM. In *A. platensis*, TMA concentration was 0.36 mM, which is around 1.0 × 10^5^ fold higher than the odour threshold. Additionally, with the exception of *T. chui*, direct extraction of TMA with MTBE allowed a larger amount of TMA to be extracted from dried biomass, in comparison to when extraction was performed after osmotic shock. Since TMA is highly soluble in water, MTBE extracted more TMA directly from the dried cells than from the aqueous solution containing the TMA following the osmotic shock.

To conclude, our study shows that the volatile profile of microalgal biomass depends greatly on the treatment the biomass underwent during harvesting and processing and also on the extraction method used to extract the volatile compounds. A uniform methodology will be necessary if different research group wish to compare volatile profiles in the same species. Moreover, improved understanding on VOCs will allow for better design of downstream processing and formulation of microalgae-based food ingredients to create appetizing food products.

## Data Availability

The raw data supporting the conclusions of this article will be made available by the authors, without undue reservation.
